# Starvation memory resulting in reproductive plasticity is conserved in some *Caenorhabditis elegans* wild isolates

**DOI:** 10.17912/micropub.biology.000243

**Published:** 2020-05-04

**Authors:** Maria C. Ow, Sarah E. Hall

**Affiliations:** 1 Syracuse University, Department of Biology, Syracuse, NY

**Figure 1:**
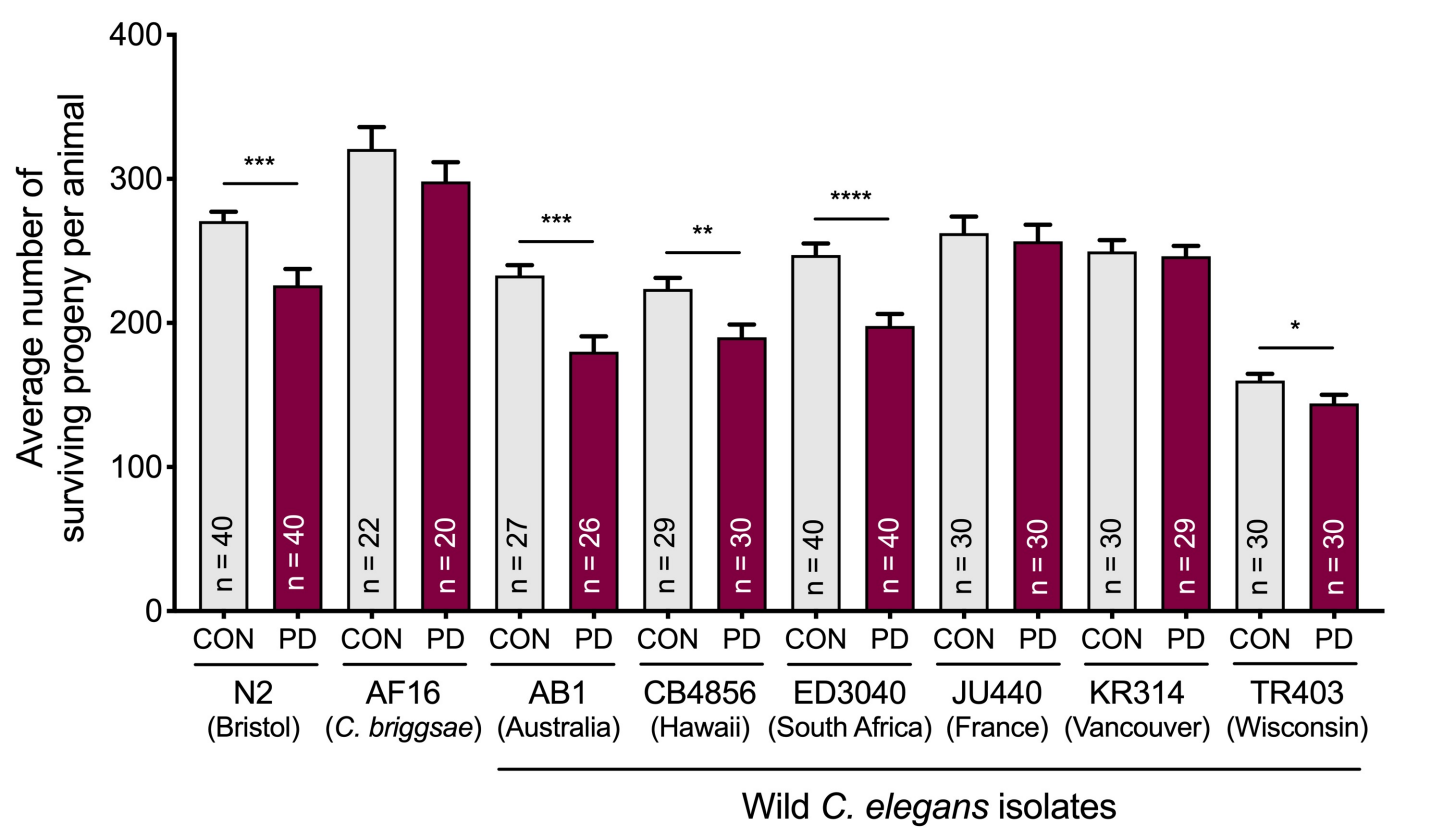
Some wild *C. elegans* isolates exhibit reproductive plasticity as a result of early life starvation. The bar graph represents the brood sizes of postdauer and control animals for *C. elegans* N2 Bristol, *C. briggsae* AF16, *C. elegans* AB1, CB4856, ED3040, JU440, KR314, and TR403. * *p* < 0.05; ** *p* < 0.01, *** *p* < 0.001, **** *p* < 0.0001 (Student’s *t*-test) indicate the brood size comparison between PD relative to CON of a given strain. Assays were performed at 20^o^C; n indicates total sample size assayed over at least three biologically independent replicates. Defects in egg laying or embryonic lethality were not quantified, but we observed no obvious differences among strains.

## Description

During its first larval stage (L1), the nematode *C. elegans* makes a critical decision regarding its developmental trajectory based on environmental conditions. Poor conditions such food scarcity, crowding, or high temperatures promote the entry into a stress-resistant, non-aging, diapause stage named dauer where they can survive for months. Upon improved conditions, dauer larvae resume development as postdauer (PD) L4 larvae and continue to reproductive adulthood as PD adults (Cassada and Russell, 1975). If conditions are favorable, L1 larvae proceed through additional larval stages (L2-L4) until reaching reproductive adulthood (control adults or CON) (Sulston and Horvitz, 1977).

We have previously shown that PD adults retain a cellular memory of their early life experience that results in stress-specific transcriptome changes that promote alterations in their life history traits (Hall *et al.* 2010, 2013; Ow *et al.* 2018). For instance, wild-type CON adults and PD adults that experienced early-life starvation exhibit reproductive differences that are manifested as decreased brood size in PD animals relative to CON adults. Reproduction in self-fertilizing hermaphrodites is sperm-limited; thus, brood size differences can result from varying numbers of sperm produced by individual animals. We showed previously that postdauers animals that experienced early-life starvation exhibit a significant delay in germline proliferation during the period when sperm are produced compared to control animals of comparable developmental stage. This observation suggests that the decrease in postdauers adult brood size is consistent with a reduction in sperm number, which we have referred to as “reproductive plasticity” (Ow *et al.* 2018).

The standard wild-type N2 strain was first cultivated in the laboratory over five decades ago, resulting in the fixation of random mutations over thousands of generations in laboratory conditions atypical to what are experienced by natural populations (Sterken *et al.* 2015). We wondered whether the reproductive plasticity observed in the canonical laboratory wild-type N2 strain is an adaptive trait acquired over time as a result of laboratory conditions, or represents a conserved mechanistic response to starvation stress. We assessed CON and PD brood sizes of six natural isolates representing various branches of the *C. elegans* phylogenetic tree (Andersen *et al.* 2012). CON L4 larvae were obtained from a continuously growing mixed population of worms cultured on NGM plates at 20^o^C. To obtain PD L4 larvae, well-fed worms were first starved on NGM plates at 20^o^C for about one week until dauers were visible, then worms were collected and incubated for 30 minutes in 1% SDS (sodium dodecyl sulfate) at room temperature with gentle rotation. Because dauers have suspended pharyngeal pumping (Cassada and Russell, 1975), they survive the 1% SDS treatment that would otherwise be lethal if the detergent were ingested. Dauers were then washed with M9 buffer to rinse away the SDS and placed onto seeded NGM plates at 20^o^C to promote dauer exit. Once the dauers had developed into postdauer L4s, brood size assays were performed in parallel with their CON counterparts by randomly singling out L4 larvae onto seeded 35 mm NGM plates at 20^o^C, transferring daily them to fresh plates until egg laying ceased, and counting the number of surviving progeny. These surviving progenies were counted as young adults (four days after the mothers were transferred to fresh NGM plates). We found that certain wild isolates, (*e.g.* AF16 in Figure 1), had the proclivity to crawl to the side of an NGM plate assay plate and desiccate. We thus censored the data for any animal that died before the end of the egg-laying period.

We found that four out of six natural isolates (AB1, CB4856, ED3040, and TR403) displayed a significant reduction in brood size in PD adults relative to CON adults similar to N2. The strains, JU440 and KR314, did not exhibit reproductive plasticity and had a similar number of progeny between CON and PD adults (Figure 1). Interestingly, when we measured the brood size in CON and PD adults of another nematode species closely related to *C. elegans*, *C. briggsae* AF16, it displayed a modest but statistically insignificant decrease in PD brood size compared to CON adults (Figure 1).

Is the reproductive plasticity between CON and PD a reflection of different mating strategies among *Caenorhabditis* strains? The spontaneous male frequency in the N2 strain is a low ~0.1%, and reproduction is usually achieved through hermaphrodite self-fertilization (Chasnov and Chow, 2002). However, we found that one natural isolate, CB4856, which harbors a higher frequency of males in their population than the laboratory N2 strain (Wegewitz *et al.* 2008), also exhibits reproductive plasticity between CON and PD. This observation suggests that reproductive plasticity between CON and PD adults may not be simply due to changes in male frequency and levels of mating.

The distinction between strains exhibiting adult reproductive plasticity and those that do not is not immediately obvious when comparing phylogenetic proximity or strain isolation date (Andersen *et al.* 2012). Thus, additional experiments would be necessary to determine the genetic loci in N2 Bristol and the wild isolates that modulate this reproductive trait. Taken together, these results suggest that reproductive plasticity observed in *C. elegans* is a naturally occurring developmental trait rather than an adaptive trait stemming from decades of cultivation in a laboratory.

## Reagents

The strains used in this study, *C. elegans* N2 Bristol, *C. briggsae* AF16, *C. elegans* wild isolates AB1, CB4856, ED3040, JU440, KR314, and TR403, were provided by the *Caenorhabditis* Genetics Center. Worms were cultured on NGM plates at 20^o^C and fed with *Escherichia coli* OP50 (Brenner, 1974). M9 buffer was prepared as described before (Stiernagle, 2006). SDS was purchased from Fisher Scientific (BP8200500).

Statistical significance between at least three biological independent brood sizes of CON adults and PD adults for a given strain was determined using a Student’s *t*-test (GraphPad Prism v.8).
